# Fc Gamma Receptor IIIB NA1/NA2/SH Polymorphisms Are Associated with Malaria Susceptibility and Antibody Levels to *P. falciparum* Merozoite Antigens in Beninese Children

**DOI:** 10.3390/ijms232314882

**Published:** 2022-11-28

**Authors:** Abdou Khadre Dit Jadir Fall, David Courtin, Rafiou Adamou, Sofie Edslev, Anita Hansen, Nadia Domingo, Michael Christiansen, Bright Adu, Jacqueline Milet, André Garcia, Michael Theisen, Florence Migot-Nabias, Célia Dechavanne

**Affiliations:** 1 Institut de Recherche pour le Développement, UMR 261 MERIT, Université Paris Cité, 75006 Paris, France; 2 Centre d’Etude et de Recherche sur les Pathologies Associées à la Grossesse et à l’Enfance, Cotonou 00229, Benin; 3 Centre for Medical Parasitology, Department of International Health, Immunology and Microbiology, Department of Infectious Diseases, University of Copenhagen, Copenhagen University Hospital, Rigshospitalet, 2300 Copenhagen, Denmark; 4 Bacteria, Parasites, and Fungi, Statens Serum Institut, Artillerivej 5, 2300 Copenhagen, Denmark; 5 Department for Congenital Disorders, Statens Serum Institut, 2300 Copenhagen, Denmark; 6 Department of Immunology, Noguchi Memorial Institute for Medical Research, University of Ghana, Legon, Accra P.O. Box LG 581, Ghana

**Keywords:** Fc gamma receptor, FcgRIIIB-NA1, -NA2, -SH variants, malaria susceptibility, antibody response, Benin

## Abstract

This paper aimed to investigate the influence of polymorphisms in the *FCGR2A* gene encoding R131H FcgRIIA variants and in the *FCGR3B* gene (108G > C, 114C > T, 194 A > G, 233C > A, 244 G > A and 316G > A) encoding FcgRIIIB-NA1, -NA2 and -SH variants on malaria susceptibility and antibody responses against *P. falciparum* merozoite antigens in Beninese children. An active malaria follow-up was conducted in infants from birth to 24 months of age in Allada, Benin. *FCGR3B* exon 3 was sequenced and *FCGR2A* exon 4 was genotyped. Antibodies directed to GLURP and MSP3 were quantified by ELISA. Association studies were performed using mixed-effect models. Individual carriage of *FCGR3B* 194 AA genotype was associated with a high number of malaria infections and a low level of IgG1 against MSP3 and GLURP-R0. High parasitemia and increased malaria infections were observed in infants carrying the FCGR3B*05 108C-114T-194A-233C-244A-316A haplotype. A reduced risk of malaria infections and low parasitemia were related to the carriages of the *FCGR3B* 108C-114T-194G-233C-244G-316A (FCGR3B*06), *FCGR3B* 108C–114T–194G–233A–244A–316A (FCGR3B*03 encoding for FcgRIIIB-SH) haplotypes and *FCGR3B* 297 TT genotype. Our results highlight the impact of *FCGR3B* polymorphisms on the individual susceptibility to malaria and antibody responses against MSP3 and GLURP in Beninese children.

## 1. Introduction

Immunity against *Plasmodium falciparum* (*Pf*) malaria is not well understood. However, it has been shown that immunoglobulin G (IgG) antibodies are major effectors of protection against *Pf* malaria [1,2,3]. IgG can act on *Pf* by agglutinating the parasites and directly preventing their reinvasion of red blood cells or indirectly by binding to Fc gamma receptors (FcgR). FcgR facilitate the engulfment of IgG-opsonized microbes, leading to the activation and regulation of the immune cell response and, at term, to parasite killing [4]. FcgR can influence the IgG binding on immune cells and impact immunity to malaria through ADCC (antibody-dependent cell-mediated cytotoxicity), ADRB (antibody dependent respiratory burst) or phagocytosis of malaria parasites [4]. FcgR are expressed at the surface of immune cells such as neutrophils [5]. Neutrophils are the most abundant leukocytes in the human blood and play key roles in innate immune response [6]. They are at the front lines of infection defenses, being generally the first circulating cells clearing pathogens including *Pf*. Phagocytosis, the production of reactive oxygen species (ROS) or other anti-microbial products [7] are described as functions that neutrophils deploy to fight malaria infection [8,9,10]. Human neutrophils constitutively express the FcgRIIA and FcgRIIIB capable of binding IgG antibodies. Various studies have investigated the relationships between polymorphisms in FcgRIIA and FcgRIIIB, IgG sub-classes and malaria immunity [11,12,13,14].

FcgRIIA binds preferentially to IgG3, and then IgG1 and IgG2, followed by IgG4 [15]. FcgRIIA initiates endocytosis, phagocytosis and the release of inflammatory mediators [16]. There are two variants for FcgRIIA, 131R (Arginine) and 131H (Histidine) which show different IgG2 binding efficiencies, being greater for the 131H variant [16]. The 131R variant has been shown to play a major role in ADRB and is associated with low phagocytic activity and poor immune complex clearance [4,17].

FcgRIIIB is one of the most abundant proteins at the surface of neutrophils, with each cell expressing between 100,000 and 200,000 copies [6,18]. It preferentially binds IgG1 or IgG3 opsonized particles [19]. FcgRIIIB is the only FcgR anchored to the cell membrane via glycosylphosphatidylinositol (GPI) and has the same number of extracellular domains as its other variant, FcgRIIIA [20,21]. The hypothesis of its synergistic effect with FcgRIIA and FcgRIIIA has been advanced by several studies, concurring to greater activate phagocytosis as well as degranulation, and consequently can better eliminate opsonized pathogens by neutrophils [22,23,24,25]. Neutrophil antigen (NA) polymorphisms are located in the membrane-distal Ig-like domain of FcgRIIIB. These polymorphisms, called human neutrophil antigens (HNAs), are HNA-1a (or NA1) encoded by the FCGR3B*01 allele, HNA-1b (or NA2) encoded by the FCGR3B*02 allele and HNA-1c (or SH) encoded by the FCGR3B*03 allele [26,27].

Other alleles (https://www.imgt.org/), as well as the absence of the FcgRIIIB molecule (NA null) for individuals whose neutrophils present a gene deletion, are described. NA polymorphisms are encoded by six nucleotides localized in the exon 3. FCGR3B*01 and FCGR3B*02 alleles differ in five 108G > C, 114C > T, 194 A > G, 244 G > A and 316G > A positions. They are both identical for the nucleotide at position 233, unlike FCGR3B*03 (rs5030738, 233C > A). This modification in FCGR3B*03 encodes for A78D, which results in the expression of the FcgRIIIB-SH allo-antigen [26,28,29]. Three additional polymorphisms of the *FCGR3B* gene were also considered in this study: 197 G > T, 297 G > T and 371 A > G, of which were previously associated with protection from malaria [30]. FcgRIIIB receptors play an important role in phagocytosis and degranulation [31]. FcgRIIIB NA1 facilitates the phagocytosis of IgG1- and IgG3-opsonized particles more efficiently than FccRIIIB-NA2 [31], while a study suggested that the 233A position of the *FCGR3B* gene could be linked to the modulation of antibody levels directed to GLURP-R0 and GLURP-R2 [32]. Regarding the other *FCGR3B* SNPs, no study focused on relationships with malaria infections.

In this study, we explored the influence of Single Nucleotide Polymorphisms (SNP) in exon 4 of *FCGR2A* and in exon 3 of *FCGR3B* in a cohort of Beninese infants followed-up for malaria from birth to 24 months of age. Among the rare literature on this topic, Adu et al. found, in Ghanaian children, an association between the carriage of the FCGR3B*03 233A allele and protection against malaria [30]. Moreover, *FCGR3B* 233AA and 233AC genotypes have been shown to modify the protective effect of both GLURP-R0 and GLURP-R2 antibodies with an important reduced risk of malaria clinical infections in Ghanaian children [32].

Our work aims to investigate the impact of these specific polymorphisms encoding FcgRIIA 131R/H, FcgRIIIB-NA1, FcgRIIIB-NA2 and FcgRIIIB-SH variants on the susceptibility to malaria infections through the number of *P. falciparum* infections and the parasite density. Since FcgRIIIB variants have also been related to the modulation of IgG concentrations [32], the role of SNPs in *FCGR3B* on levels of IgG1, IgG2 and IgG3 specific to MSP3 and GLURP-R0 and GLURP-R2 antigens was also investigated.

## 2. Results

### 2.1. Characteristics of Study Participants

As presented in Table 1, 345 Beninese infants were followed-up for 18 months. Out of 345, 280 (81%) infants presented at least one *P. falciparum* infection with a mean of 4.44 infections per infant and a maximum of 16 infections for one child. Sixty-five (19%) infants had no *P. falciparum* infection. Infants belonged mostly to the Aïzo ethnic group. Differences appeared between Aïzo, Fon and other ethnic groups regarding malaria infection (*p* = 0.041). Infants from the non-infected group were from a slightly better socio-economic environment (*p* = 0.098). There were no differences between infected and non-infected infants regarding sex, birth weight and bednet use. The majority of the mothers were married (98%), young (25 years, in mean), had two children on average and no *P. falciparum* infections during pregnancy (77% and 69% in the groups of non-infected and infected infants, respectively). Finally, infants from the non-infected group were less exposed to mosquitoes than infants from the infected group (*p* = 0.0001).

### 2.2. Associations between FCGR2A SNP, FCGR3B SNPs, and Malaria Infections or Parasite Density

Table 2 shows the observed distribution of *FCGR2A* and *FCGR3B* genotypes in the whole study group. None of the FcgRIIA 131RH, 131RR nor 131HH genotypes were associated with malaria infections or with parasite density (Table 3A). This result was confirmed by using the allelic model (Table 3A).

Among the *FCGR3B* SNPs studied, the results showed a high number of malaria infections associated with the carriage of *FCGR3B* 194AA (*p* = 0.027), while the *FCGR3B* 194 G-allele was related to a low risk of infection (*p* = 0.029) (Table 3B). In contrast, the carriage of *FCGR3B* 297TT was related to protection against malaria infections through a decrease in both the number of infections and level of parasitemia (*p* = 0.018 and *p* = 0.050, respectively) (Table 3B).

### 2.3. Associations between FCGR3B-Combined SNPs and Malaria Infection or Parasite Density

In this section, the influence on malaria infections of possible *FCGR3B*-combined SNPs, including those encoding the FcgRIIIB-NA1, -NA2 and -SH variants (108G > C, 114C > T, 194 A > G, 233C > A, 244 G > A, 316 G > A), was studied.

**Figure 1:**
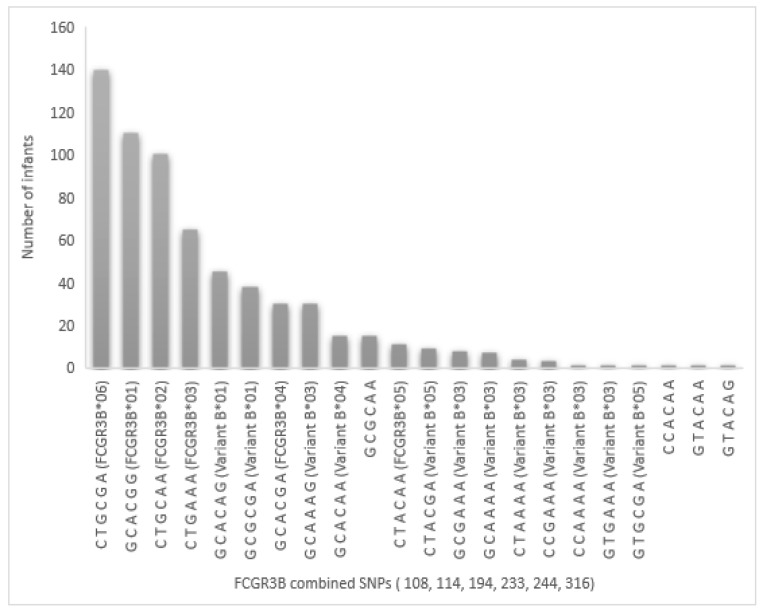
Distribution of the *FCGR3B* SNP combinations among the study group. SNP combinations are listed in increasing order of their prevalence in the study group (n = 318 children and N = 636 combinations of alleles). The combination of SNPs are named according to the IMGT^®^ database (https://www.imgt.org/IMGTrepertoireRPI/Proteins/tables/index.php?species=human&gene=FCGR3B#notes, accessed on 8 September 2022). The variant, FCGR3B*01, is also called [FcgRIIIB-NA1], and the same applies for FCGR3B*02 [FcgRIIIB-NA2] and FCGR3B*03 [FcgRIIIB-SH].

The *FCGR3B* SNP combinations observed in the study group are presented in Figure 1. The combination 108C-114T-194G-233C-244G-316A (FCGR3B*06) was prevalent in the study population at 22%. Of note, 22 sequences were found in this population, whereas only 12 were described in the IMGT^®^ database.

Only the haplotype 108C-114T-194A-233C-244A-316A (that differs from FCGR3B*02 only in 194 A > G) was associated with both high numbers of infections and high levels of parasitemia (*p* = 0.011 and *p* = 0.0001, respectively) (Table 4). In contrast, 108C-114T-194G-233C-244G-316A (FCGR3B*06) was the unique combination of *FCGR3B* SNPs associated with both low numbers of infections and low levels of parasitemia (*p* = 0.018 and *p* = 0.043, respectively). The results in Table 4 also showed a protective effect against *P. falciparum* infections in relation to the carriage of FcgRIIIB-SH haplotype (FCGR3B*03, *p* = 0.035).

### 2.4. IgG Isotype Levels to P. Falciparum Merozoite Antigens, FCGR3B SNPs, and FCGR3B-Combined SNPs

**Figure 2:**
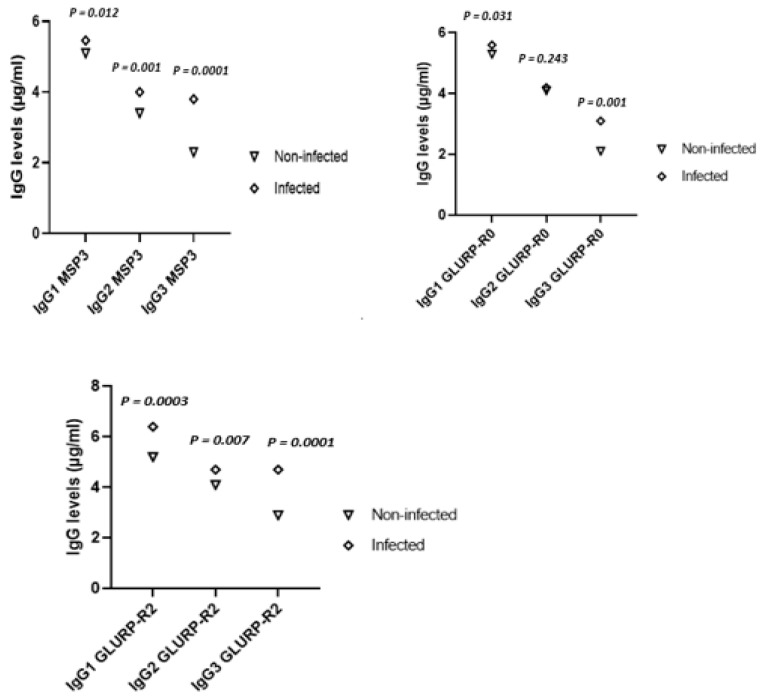
Comparison of the level of IgG against MSP3 (Merozoite Surface Protein 3), GLURP-R0 (Glutamate Rich Protein R0) and GLURP-R2 (Glutamate Rich Protein R0) *P. falciparum* antigens according to a malaria infection. These figures present the distribution of IgG levels (median) to *P. falciparum* merozoite antigens at 24 months between the infected and non-infected groups. Statistical significance was determined using the Mann–Whitney *U*-test. *P*-values were compared between the non-infected and infected groups.

As expected, the group of infants exposed to a *Pf* infection had higher concentrations of IgG against the candidate vaccine antigens than the group of non-infected infants, except for IgG2 against MSP3 and GLURP-R0 (Figure 2).

**Figure 3:**
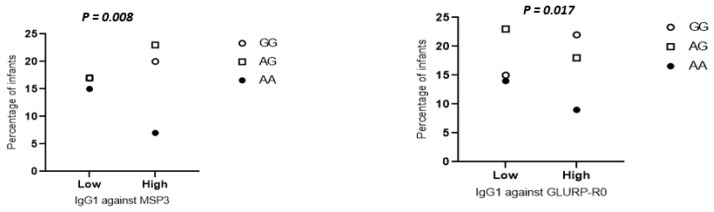
IgG1 levels of infants to MSP3 (Merozoite Surface Protein 3) and GLURP-R0 (Glutamate Rich Protein R0) according to *FCGR3B 194 A/G* genotypes. Anti-merozoite IgG1 data were categorized into two levels, low and high, according to the median. The IgG levels at 24 months were used. Statistical significance was determined by the Kruskal–Wallis test. Only significant associations are shown.

IgG levels to MSP3, GLURP-R0 and GLURP-R2 recombinant antigens were dichotomized into low and high according to the median value. When taking into account the individual carriage of *FCGR2A* and *FCGR3B* polymorphisms, only two associations emerged, involving the *FCGR3B* 194AA genotype, which was associated with a low level of IgG1 against GLURP-R0 and MSP3 compared to *FCGR3B* 194GG (*p*-value = 0.017 and 0.008, respectively) (Figure 3).

**Figure 4:**
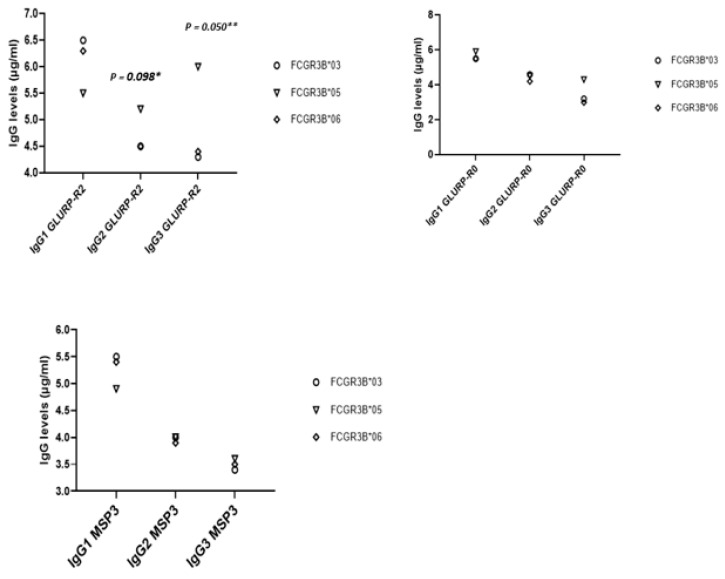
Relationship between IgG levels at 24 months of age, and significant FCGR3B-combined SNPs. These figures present the distribution (median) of IgG1, IgG2 and IgG3 levels (at 24 months) to *P. falciparum* merozoite antigens in the case of the FCGR3B-combined SNPs found to be associated with malaria phenotypes. Statistical significance was determined by the Mann–Whitney test. Only significant or significant trend associations are shown. * Trend of significant *P* value at *p*-value ≤ 0.10: IgG levels between the carriage of FCGR3B*05 and FCGR3B*06 (or FCGR3B*03). ** significant *p*-value (*p*-value ≤0.05): IgG levels between the carriage of FCGR3B*05 and FCGR3B*06 (or FCGR3B*03).

According to Figure 4, infants carrying FCGR3B*05 had higher IgG3 levels against GLURP-R2 (*p* = 0.050) compared to infants carrying FCGR3B*06 and *03. The same pattern was observed for IgG2 against GLURP-R2—even the *p*-value was not significant at *p* = 0.05 (0.098). Infants carrying FCGR3B*06 and *03 had higher IgG1 levels against MSP3 and GLURP-R2 compared to infants carrying FCGR3B*05—even the differences were not significant.

## 3. Discussions

This study focused on the impact of specific polymorphisms encoding FcgRIIA 131 R/H and FcgRIIIB-NA1/-NA2/-SH variants on the susceptibility to malaria infection through the number of infections and parasite density. Genetic polymorphisms present in *FCGR2A* and *FCGR3B* genes could alter the affinity of FcgRIIA and FcgRIIIB receptors for IgG, and therefore, influence the quality of the immune response against malaria. No association between malaria phenotypes and FcgRIIA 131 R/H (rs1801274) variants was found in Beninese children from Allada. This result is consistent with findings from another study previously performed in Benin in the Tori Bossito area [14]. It may be explained by the fact that FcgRIIA needs to interact with other FcgR, particularly FcgRIIIB. Indeed, these receptors have been shown to exhibit synergistic responses: FcgRIIA is essential for the induction of efficient effector functions, while a high abundance of FcgRIIIB may guarantee an efficient interaction with IgG complexes [33].

Among the SNPs located in exon 3 of *FCGR3B*, we found that the *FCGR3B* 194AA genotype was associated with an increased risk of malaria infection, which may be explained by its association with low IgG1 levels to MSP3 and GLURP-R0. In other studies, high IgG levels to MSP3 and GLURP antigens have been associated with malaria protection [33,34,35]. Moreover, unlike the *FCGR3B* 194 G-allele, for which we had an association with a low risk of malaria infection, the *FCGR3B* 194 A-allele may alter the affinity of IgG sub-classes for the receptor. Indeed, it is known that the *FCGR3B* 194 G-allele encodes for a Serine (S) at position 65 of FcgRIIIB. In combination with the Asparagine (N) at position 63, the *FCGR3B* 194 G-allele leads to a glycosylation site. Based on our observation, we hypothesize that the loss of glycosylation due to the *FCGR3B* 194 A-allele may decrease the immune activation of neutrophils against *P. falciparum* [36]. This form could even be related to a poorer ingestion of IgG1 or IgG3 opsonized particles [31]. In line with this, we also observed that the haplotype 108C-114T-194A-233C-244A-316A (FCGR3B*05) was associated with a high risk of malaria infection and parasite density. Therefore, the association observed between malaria susceptibility and this haplotype could result from the presence of the A-allele at position 194 of *FCGR3B*.

In our study, no association was found between the *FCGR3B* 233 A-allele and malaria protection, whereas we observed that the carriage of 108C-114T-194G-233A-244A-316A encoding for the FcgRIIIB-SH haplotype was related to a decreased risk of malaria infection. This observation is in line with the results of Adu et al. [30], who found an association between FcgRIIIB-SH and protection against clinical malaria in Ghanaian children.

The FcgRIIIB-SH has been also implicated in a higher expression of the FcgRIIIB receptor [37]. Since FcgRIIIB plays an important role in phagocytosis and degranulation, this form could be associated with the better ingestion of IgG1 or IgG3 opsonized particles [31] or other mechanisms involved in the immune response against malaria, such as the ADRB [4]. *FCGR3B* 233 A/C has been shown to potentially modulate antibody concentrations to GLURP-R0 and GLURP-R2 [32]. Children carrying *FCGR3B* 233AA and 233AC genotypes showed a reduced risk of clinical malaria in Ghana [32]. The *FCGR3B* 233 A-allele encodes for a substitution of the hydrophobic amino acid alanine (A) by the negatively charged aspartic acid (D) at position 78. It has been proposed that this substitution improved the binding of IgG, and therefore, may lead to malaria protection [30,37].

Our results showed associations between the *FCGR3B* 297TT genotype and a decreased risk of *P. falciparum* infections and low parasitemia. Adu et al. found the 297 T-allele to be significantly associated with protection from clinical malaria through a Fisher test [30]. These results are interesting since only two studies investigating its correlation with malaria susceptibility found the same results. In addition, our study correlated this allele with the level of parasitemia. The *FCGR3B* 297 T-allele may favor an important biological mechanism by enhancing the capability of IgG antibodies to trigger neutrophil-mediated functions such as opsonized merozoite phagocytosis. The influence of the *FCGR3B* 297 T-allele deserves further exploration in order to better understand the underlying mechanisms and to evaluate its impact on malaria protection.

Finally, the 108G > C, 114C > T, 194A > G, 244G > A and 316G > A allele distribution encoding the NA1/NA2 system revealed significant deviations from the HWE expectation. This result was in line with our previous observation regarding the distribution of NA1/NA2 genotypes in a Beninese population [14] and in Kenyan children [38,39]. The HWE deviation observed for *FCGR3B* could be due to consanguinity [39]—unidentified mutations likely resulting from disease-related evolutionary selection pressure exerted by *P. falciparum* and potentially by other infectious diseases occurring in the population that do not affect the neighboring *FCGR2A* and *FCGR3B* genes.

In conclusion, the present study showed that the *FCGR3B* 194AA genotype was associated with a higher risk of malaria infection and low IgG levels to GLURP-R0 and MSP3, while the *FCGR3B* 297 T-allele was associated with a reduced risk of *P. falciparum* infections and low parasitemia. Moreover, a higher risk of malaria infection and parasitemia was related to the *FCGR3B* 108C-114T-194A-233C-244A-316A (FCGR3B*05) haplotype.

This study provides a justification for a more detailed functional characterization of the polymorphisms located at positions 194 and 297 of the *FCGR3B* gene. Additionally, further studies are needed for a deeper understanding of the mechanisms involved in the relationships observed between gene polymorphisms and specific antibody levels.

## 4. Materials and Methods

### 4.1. Study Area and Design

The study was conducted in the district of Allada (southern Benin), in a semi-rural area where *Pf* malaria is hyperendemic. This study was part of the TOLIMMUNPAL project (“Tolerance Immunitaire et Paludisme”) which was conducted from January 2010 to December 2011 with 345 newborns enrolled and followed-up from birth to 24 months of age.

This follow-up consisted of epidemiological, environmental and parasitological monitoring. Routine parasitological monitoring was performed monthly using thick blood smears (TBS) to detect asymptomatic infection. In case of fever, both a rapid diagnostic test (RDT) and a TBS were carried out. Symptomatic malaria (fever or history of fever in the preceding 24 h and positive RDT and/or TBS) was treated according to the recommendations of the national malaria control program. Parents were invited to bring their infants to the two health centers of reference (Attogon and Sekou) at any time in the eventual suspicion of malaria, fever or clinical signs related to malaria or not. To assess the environmental risk of malaria exposure, environmental (house characteristics, surroundings, mosquito captures) and geographical (satellite images, soil type, watercourse nearby, vegetation index, rainfall) data were recorded. This allowed for modeling, for each child included in the follow-up, an individual risk of exposure to *Anopheles* bites by means of a space- and time-dependent variable [40].

### 4.2. FcgRIIA Genotyping

Genetic polymorphisms encoding for FcgRIIA 131R/H were identified using a polymerase chain reaction restriction fragment length polymorphism (PCR-RFLP) method described by Jiang et al. [41]. *FCGR2A* contains a non-synonymous variant (c.494G/A, rs1801274), where a G-to-A substitution in exon 4 changes an amino acid from arginine (R, codon CGT) to histidine (H, codon CAT) in residue 131 of the protein. The PCR-RLFP method used the sense primer (5′-GGAAAATCCCAGAAATTCTCGC-3′) and the antisense primer (5′-CAACAGCCTGACTACCTATTACGCGGG-3′) [30]. The sense primer ends immediately 5′ to the polymorphic site in codon 131, and contains a nucleotide substitution (underlined), which introduces a BstUI restriction enzyme site (5′-CGCG-3′) into the PCR product when the next nucleotide is G. The antisense primer is located in the downstream intron and contains two nucleotide substitutions (underlined), which introduce an obligate BstUI site into all PCR products. This serves as an internal control for successful restriction enzyme digestion. During BstUI digestion, infant DNA containing the CGT codon is cut twice (generating a 322 bp DNA fragment) and infant DNA containing the CAT codon is cut once (generating a 343 bp DNA fragment). The final BstUI restriction digestion products were visualized as 343 bp (HH genotype), 343 and 322 pb (RH genotype) and 322 bp (RR genotype) bands on 3% agarose with ethidium bromide staining.

### 4.3. Sequencing of FCGR3B Exon 3

Sequencing *FCGR3B* exon 3 required a multistep procedure to be sure that only exon 3 from *FCGR3B* was sequenced due to a high similarity of the sequences of exon 3 from *FCGR3B* and *FCGR3A*. First of all, a long-range PCR, amplifying a larger segment of *FCGR3B* (exon 1 to intron 3), was first carried out. In this, 10 ng/μL of infant DNA was amplified used a 20 mM sense primer (5′-CTCCATTGGGAGACTTGAGAT-3′) and a 20 mM antisense primer (5′-CGTGGTTTCTAAGGTGTCACAGG-3′) [30]. Then, the amplified DNA segments were separated by electrophoresis on a 1% agarose gel containing ethidiumbromid, and then were extracted by a gel extraction kit. For the first step, 5 μL of DNA loading buffer was added to each sample of long-range PCR products (15 μL/well). A blank sample acting as a control, and a 7 μL DNA marker, were also loaded to the gel. The electrophoresis was run at 90V for 45 mn. Gel pieces containing the DNA bands, visualized in a UV transluminator at 312 nm, were cut out of the gel and transferred to 1.5 mL eppendorf tubes (one sample/tube). The tubes were stored overnight at 5 °C. The extracted DNA was used in a nested PCR, where exon 3 from *FCGR3B* was amplified. Amplicons were used in a nested PCR to amplify exon 3 of *FCGR3B* with the M13 tagged sense (5′-tgtaaaacgacggccagtCTCAGCTTCATGGTCTTGGATTG-3′) and antisense (5′-caggaaacagctatgaccACACATTCACATTGTATGCACTCCA-3′) primers. The quality of the nested PCR products was checked by gel electrophoresis before the final stage of the sequencing reaction. The amplified DNA was then replicated in a sequencer reaction where fluorescent-labeled dideoxynucleotides (ddNTPs) were added to the M13 primers. The four ddNTPs were labeled with fluorescent dyes with different wavelengths, thus, giving different signals when measured in a DNA Analyzer machine. The *FCGR3B* exon 3 sequences were analyzed with the software program Sequencher 5.1 (Gene Codes Corporation) [30].

### 4.4. ELISA Assay

The Enzyme-Linked Immunosorbent Assay (ELISA) standard operating procedures developed by the African Malaria Network Trust were used to assess the concentrations of IgG1, IgG2 and IgG3 specific to the MSP3/FC27 strain and GLURP/F32 strain vaccine candidate antigens for malaria. MSP3 (amino acids 212–380, F32 strain), GLURP-R0 (amino acids 25–514, F32 strain) and GLURP-R2 (amino acids 706–1178, F32 strain) were obtained through *E. coli* [13]. The protocol for the IgG quantification was previously described elsewhere [13].

Briefly, plasma samples (from infants at 6, 12, 15, 18 and 24 months of age) were diluted in a dilution buffer (1% milk powder in 1X PBS 0.1% Tween 20, 0.02% NaAz) 1∶50 for IgG1, IgG2, IgG3 to MSP3 and GLURP. Horse radish peroxidase-conjugated anti-IgG1 (clone NL16, Skybio, Bedfordshire, UK) at 1:2000 dilution as well as anti-IgG2 (clone HP 6002, Sigma, Saint-Louis, MO, USA) and anti-IgG3 (clone ZG4, Skybio) at 1:5000 dilution were used as detection antibodies. The plates were washed thrice with PBS Tween-20 (0.1%) NaCl (0.5 M) after blocking and incubation with primary and detection antibodies. ADAMSEL software (Auditable Data Analysis and Management System for ELISA) was used to transform optical density (OD) values into antibody concentrations [13]. IgG1, IgG2 and IgG3 against *Pf* merozoite antigens were quantified at 6, 12, 15, 18 and 24 months of age, but the analysis used the levels at 24 months.

### 4.5. Statistical Analysis

The clinical phenotypes of interest were the number of *P. falciparum* infections per infant during the follow-up and the parasite density. The parasite density corresponded to the number of parasites per microliter of blood (based on 8000 leukocytes/μL). Chi-square and Mann–Whitney tests were used to compare the demographic and clinical characteristics between infected and non-infected groups.

The genotypic frequencies of *FCGR2A* and *FCGR3B* haplotypes were tested for the Hardy–Weinberg equilibrium (HWE). Association analyses between malaria infections during the 24-month follow-up and *FCGR2A* and *FCGR3B* polymorphisms were tested using a mixed-effects Poisson regression, while associations with parasite density were tested using a mixed-effects linear regression model.

First of all, the influence of the carriage of *FCGR2A* and *FCGR3B* SNPS and genotypes on the clinical phenotypes was studied. For each polymorphism, the reference was the homozygote with the highest number. The carriage of specific alleles was even studied. Then, we established the possible *FCGR3B*-combined SNPs, including those encoding the FcgRIIIB-NA1, -NA2 and -SH variants (108G > C, 114C > T, 194 A > G, 233C > A, 244 G > A and 316G > A); their associations with malaria infections were studied.

The models were adjusted using covariates listed in Table 1. Univariate regression analyses on each of the covariates were made and only those with a *p*-value < 0.20 in the univariate model were included in the mixed-effects models. The mother’s age (in years), child’s age (in months), sex, birth weight, ethnic group, socio-economic score, number of infants per mother, infection during pregnancy, mosquito exposure and bednet use were used according to the criteria.

Finally, the levels of IgG1, IgG2 and IgG3 against MSP3, GLURP-R0 and GLURP-R2 at 24 months of age were investigated for each SNP or SNP combinations.

Statistical analysis was carried out using the Stata software version 14.

## Figures and Tables

**Table 1:** Characteristics of participants.

Characteristics of the Study Group	At Least One *P. Falciparum* Infection in Infants *		
	No (n = 65)	Yes (n = 280)	***p*-Value**
**Mothers**			
Married (n, %):	Yes: 64 (98.46) No: 1 (1.53)	Yes: 275 (98.21) No: 5 (1.78)	0.893 ^a^
Job (n, %):	Yes: 32 (49.23) No: 33 (50.77)	Yes: 118 (42.14) No: 162 (57.85)	0.298 ^a^
Number of infants, (median, Q1-Q3)	2 (1–3)	2 (1–4)	0.071 ^a^
Mother’s age (median, Q1-Q3)	25 (22–28)	25 (21.5–30)	0.116 ^a^
Infection during pregnancy (n, %):	Yes: 15 (23.07) No: 50 (76.92)	Yes: 87 (31.07) No: 193 (68.92)	0.203 ^a^
**Infants**			
Birth weight (g, median, Q1-Q3)	2950 (2710–3190)	3030 (2810–3300)	0.216 ^a^
Sex (n, %): Male Female	36 (55.38) 29 (44.61)	147 (52.5) 133 (47.5)	0.673 ^a^
Ethnic group (n, %): Aïzo Fon Others	42 (73.84) 12 (18.46) 11 (16.92)	195 (69.64) 65 (23.21) 20 (7.14)	**0.041** ^a^
*P. falciparum* infections in infants: Mean number of infections Min-Max Parasite density (mean nb parasites/µL)	0	4.44 1–16 9105.77	**0.0001 ^a^**
Socio-economic Score ^c^ (mean ± SD)	1.692 (1.08)	1.646 (1.06)	0.098 ^a^
Bednet use score ^b^ (mean of use ± SD)	3.62 (0.84)	3.51 (0.86)	0.315 ^a^
Mosquito exposure (med of exposure, Q1–Q3)	0.9 (0.6–1.2)	1 (0.7–1.5)	**0.0001 ^a^**

This table presents the characteristics of the group of infants according to the presence or not of at least one *P. falciparum* infection (* at least 18th months of follow-up). ^a^ Statistical significance determined using Mann-Whitney *U*-test or χ2 analysis. ^b^ Bednet use score: use of bednet defined from the proportion of “yes” to the question “Did the child sleep the night before under the net?”); 1/rare use, 2/infrequent use, 3/frequent use and 4/systematic use. ^c^ Socio-Economic score: coded from 0 to 4 corresponding to the sum of the responses 1/yes, 0/no for the following 4 indicators: electricity within the household; possession of a refrigerator, possession of a television set, possession of a two-wheeler. In bold: significant *p* value at the 0.05 threshold.

**Table 2:** Distribution of *FCGR2A* and *FCGR3B* genotypes in the study group.

Genetic Modification	Variation	Protein Variant	Genotype (Haplotype) n (%)			Minor Allele Frequency (%)	HWE Test ^a^ (*p* Value)
*FCGR2A* 494 A/G	rs1801274	R131H	HH 58 (18.98)	RH 163 (53.09)	RR 86 (27.73)	H (45)	1.52 (0.21)
*FCGR3B*							
108 G/C	rs403016	R36S	GG [NA1] 83 (26.01)	GC 137 (42.94)	CC [NA2/SH] 99 (31.03)	G (47)	6.15 (0.01) *
114 C/T	rs447536	synonymous coding (L38L)	CC [NA1] 82 (25.78)	CT 139 (43.71)	TT [NA2/SH] 97 (30.05)	C (47)	4.88 (0.02) *
194 A/G	rs448740	N65S	AA [NA1] 66 (20.68)	AG 134 (42.00)	GG [NA2/SH] 119 (37.30)	A (42)	5.90 (0.01) *
197 G/T	rs374112391	R66L	GG 5 (1.56)	GT 49 (15.36)	TT 265 (83.07)	G (9)	2.29 (0.12)
233 C/A	rs5030738	A78D	CC [NA1/NA2] 217 (62.35)	CA 83 (23.85)	AA [SH] 18 (5.17)	A (19)	6.41 (0.01) *
244 A/G	rs147574249	N82D	GG [NA1] 101 (31.66)	AG 127 (39.81)	AA [NA2/SH] 91 (28.52)	A (48)	13.14 (<0.01) *
297 G/T	rs368410676	synonymous coding (P99P)	GG 292 (91.53)	GT 22 (6.89)	TT 5 (1.56)	T (5)	24.33 (<0.01) *
316 A/G	rs2290834	I106V	GG [NA1] 37 (11.59)	GA 113 (35.42)	AA [NA2/SH] 169 (48.42)	G (29)	6.72 (<0.01) *
371 A/G	rs1373400409	H124R	AA 53 (16.61)	GA 130 (40.75)	GG 136 (42.63)	A (37)	5.04 (0.02) *

^a^ Hardy–Weinberg equilibrium test based on genotypic frequency results from the children’s DNA (n = χ2 (df = 2) * Significant *p*-value at 0.05 (*p*-value ≤ 0.05). Significant *p*-value indicated that there was deviation from HWE. Sample numbers varying from 307 (FcgRIIA 131R/H) to 318 (*FCGR3B* 114C>T and 233 C>A) and to 319 (other SNPs). Six polymorphisms of the *FCGR3B* gene (108G > C, 114C > T, 194 A > G, 233C > A, 244 G > A and 316G > A) encode for the FcgRIIIB-NA1, -NA2 and -SH variants.

**Table 3:** Association between FcgRIIA 131 R/H and *FCGR3B* single SNPs and malaria infections. (**A**): FcgRIIA 131 R/H (c.497 G/A) and malaria infections. (**B**): *FCGR3B* 194 A > G, 297 G > T and malaria infections.

**(A)**				
	**n**	**IRR**	**CI 95%**	***p*-Value**
**Number of *P. falciparum* infections**				
**Genotypes**				
**RR**	86			
**RH**	163	0.89	0.73; 1.09	0.272
**HH**	58	0.94	0.73; 1.21	0.649
**Alleles**				
**R**		0.95	0.76; 1.19	0.712
**H**		0.92	0.76; 1.12	0.436
***P. falciparum* parasite density**				
**Genotypes**				
**RR**	86			
**RH**	163	798.43	−2662.13; 4259.00	0.651
**HH**	58	−141.44	−4602.44; 4319.54	0.950
**Alleles**				
**R**		289.88	−3357.67; 3937.45	0.876
**H**		669.48	−2449.99; 3788.96	0.674
**(B)**				
	**n**	**IRR**	**CI 95%**	***p*-Value**
**Number of *P. falciparum* infections**				
**Genotypes**				
***FCGR3B* 194 A/G**				
**GG**	119			
**AG**	134	1.05	0.87; 1.28	0.553
**AA**	**66**	**1.28**	**1.02; 1.61**	**0.027**
***FCGR3B* 297 G/T**				
**GG**	292			
**GT**	22	0.73	0.50; 1.06	0.100
**TT**	**5**	**0.46**	**0.21; 1.00**	**0.050**
**Alleles**				
***FCGR3B* 194 A/G**				
**A**		1.13	0.95; 1.35	0.157
**G**		**0.80**	**0.65; 0.97**	**0.029**
***FCGR3B* 297 G/T**				
**G**		2.06	0.95; 4.47	0.067
**T**		**0.66**	**0.47; 0.93**	**0.018**
**Parasite density**				
**Genotypes**				
***FCGR3B* 297 G/T**				
**GG**	292			
**GT**	22	−3984.73	−9927.02; 1957.55	0.189
**TT**	5	−8874.19	−19,390.84; 1642.45	0.098
**Alleles**				
***FCGR3B* 297 G/T**				
**G**		8656.59	−1879.81; 19,193.01	0.100
**T**		**−5134.44**	**−10,382.69; 113.79**	**0.050**

**(A)** presents the mixed-effects Poisson regression model (number of *P. falciparum* infections) and the mixed-effects linear regression model (parasite density) obtained through the control variables of mother’s age (in years), child’s age (in months), sex, birth weight, ethnic group, socio-economic score, number of infants per mother, infection during pregnancy, mosquito exposure and bednet use. In bold: significant *p*-value ≤ 0.05 threshold. In the allelic model, the influence of the carriage of specific alleles is studied. **(B)** presents the mixed-effects Poisson regression model (number of *P. falciparum* infections) and the mixed-effects linear regression model (parasite density) obtained through the control variables of mother’s age (in years), child’s age (in months), sex, birth weight, ethnic group, socio-economic score, number of infants per mother, infection during pregnancy, mosquito exposure and bednet use. In bold: significant *p*-value ≤ 0.05 threshold. In the allelic model, the influence of the carriage of specific alleles is studied. Only significant (in bold) or significant trend (underlined) associations (at *p*-value ≤ 0.05) among FCGR3B SNPs are shown.

**Table 4:** *FCGR3B*-combined SNPs, malaria infections and parasite density.

**(A)**				
*FCGR3B* combined polymorphisms				
Number of infections	**n**	**IRR**	**CI 95%**	***p*** **-Value**
FCGR3B*05 108C–114T–194A–233C–244A–316A	11	2.44	1.23; 4.86	**0.011**
FCGR3B*06 108C–114T–194G–233C–244G–316A	140	0.58	0.37; 0.91	**0.018**
Parasite density		**Coef.**	**CI 95%**	***p*** **-Value**
FCGR3B*05 108C–114T–194A–233C–244A–316A	11	29,699.73	16,242.34; 43,157.13	**0.0001**
FCGR3B*06 108C–114T–194G–233C–244G–316A	140	−10,373.26	−20,409.75; −336.7668	**0.043**
**(B)**				
Number of infections	**n**	**IRR**	**CI 95%**	***p*** **-Value**
FCGR3B*03 108C–114T–194G–233A–244A–316A	65	0.53	0.29; 0.95	**0.035**

**(A)** presents the *FCRG3B*-combined SNPs, for which only significant associations were found. A mixed-effects Poisson regression was used for the number of infections and a mixed-effects linear regression for the parasite density. The control variables of mother’s age (in years), child’s age (in months), sex, birth weight, ethnic group, socio-economic score, number of infants per mother, infection during pregnancy, mosquito exposure and bednet were used for the two models. **(B)** presents the mixed-effects Poisson regression for the number of infections regarding the carriage of FCGR3B*03 (encoding for FcgRIIIB-SH). The control variables of mother’s age (in years), child’s age (in months), sex, birth weight, ethnic group, socio-economic score, number of infants per mother, infection during pregnancy, mosquito exposure and bednet were used for the two models. In bold: significant *p*-value ≤ 0.05.

## Data Availability

The datasets used and/or analysed during the current study are available from the corresponding author on reasonable request.
